# Current status of carbon monoxide dehydrogenases (CODH) and their potential for electrochemical applications

**DOI:** 10.1186/s40643-023-00705-9

**Published:** 2023-11-27

**Authors:** Rebecca Bährle, Stefanie Böhnke, Jonas Englhard, Julien Bachmann, Mirjam Perner

**Affiliations:** 1 Department of Marine Geomicrobiology, Faculty of Marine Biogeochemistry, GEOMAR Helmholtz Centre for Ocean Research Kiel, Wischhofstr. 1-3, 24148 Kiel, Germany; 2 Chemistry of Thin Film Materials, IZNF, Friedrich-Alexander-Universität Erlangen-Nürnberg, Cauerstr. 3, 91058 Erlangen, Germany

**Keywords:** CO_2_ fixing microorganisms, Carbon monoxide dehydrogenase (CODH), CO_2_ reduction, Electrocatalysis, Biocatalysts

## Abstract

Anthropogenic carbon dioxide (CO_2_) levels are rising to alarming concentrations in earth’s atmosphere, causing adverse effects and global climate changes. In the last century, innovative research on CO_2_ reduction using chemical, photochemical, electrochemical and enzymatic approaches has been addressed. In particular, natural CO_2_ conversion serves as a model for many processes and extensive studies on microbes and enzymes regarding redox reactions involving CO_2_ have already been conducted. In this review we focus on the enzymatic conversion of CO_2_ to carbon monoxide (CO) as the chemical conversion downstream of CO production render CO particularly attractive as a key intermediate. We briefly discuss the different currently known natural autotrophic CO_2_ fixation pathways, focusing on the reversible reaction of CO_2_, two electrons and protons to CO and water, catalyzed by carbon monoxide dehydrogenases (CODHs). We then move on to classify the different type of CODHs, involved catalyzed chemical reactions and coupled metabolisms. Finally, we discuss applications of CODH enzymes in photochemical and electrochemical cells to harness CO_2_ from the environment transforming it into commodity chemicals.

## Introduction

Since the start of the industrial revolution, carbon dioxide (CO_2_) levels in the atmosphere have increased dramatically (from 278 ppm pre-industrial to currently 417 ppm) (Rudd 2022). CO_2_ absorbs and radiates heat and is the most important greenhouse gas. The oceans are the greatest ally against human-induced climate change as they have taken up about 26% of the total anthropogenic CO_2_ emissions and captured most of the excess heat (Fox-Kemper 2021; Friedlingstein et al. 2022). The oceanic CO_2_ and heat capture, however, have promoted ocean acidification and deoxygenation (Schmidtko et al. 2017; Brauko et al. 2020). This is having detrimental effects on earth’s ecosystem functioning (Henson et al. 2017; Bates and Johnson 2020; Jin and Gao 2021; Viitasalo and Bonsdorff 2022). Particularly affecting oceanic biodiversity, productivity, and biogeochemical cycling (Brauko et al. 2020) and consequently impacting the world’s economy. To significantly reduce atmospheric CO_2_ concentration and counteract climate change and its consequences, CO_2_ emissions must be actively reduced. It is well-known that a significant mitigation of anthropogenic CO_2_ emissions alone is not sufficient (Fawzy et al. 2020). However, a broad range of alternative and innovative techniques involving CO_2_ capture, conversion, and storage could offer a viable solution. Indeed, in recent decades, various research approaches have been carried out to convert CO_2_ into sustainable commodities, such as syngas, methanol, acetate, polymers and biofuels using biotransformation and catalytic properties (Tirado-Acevedo et al. 2010; Liew et al. 2022; Akash et al. 2023).

Microbes are phylogenetically and metabolically highly diverse (Kennedy et al. 2007; Fuhrmann 2021) and have been sequestering CO_2_ naturally for millions of years (Berg 2011; Kajla et al. 2022). The use of their biocatalysts (enzymes) offers numerous advantages. Compared to conventional electrochemical conversions, biocatalysts can target chemical reactions highly specifically, be very efficient and produce “clean” products, i.e., no toxic side compounds (Schlager et al. 2017b; Fukuyama et al. 2020). Microbes have evolved at least seven autotrophic carbon fixation pathways (Hügler and Sievert 2011; Bierbaumer et al. 2023) and it can be expected that a larger set of autotrophic mechanisms are hidden among the uncultured microbial majority, suggestive by the fact that only recently three novel pathways have been proposed (Santos Correa et al. 2023). The different CO_2_ fixing enzymes help drawing down anthropogenically generated CO_2_. Current research is focusing on how to improve the microbial CO_2_ fixation ability by, e.g., creating new synthetic pathways (Schwander et al. 2016) and converting CO_2_ into valuable feedstocks, such as acetate (Liew et al. 2022).

## Microbial autotrophic CO_2_ fixation pathways

**Fig. 1:**
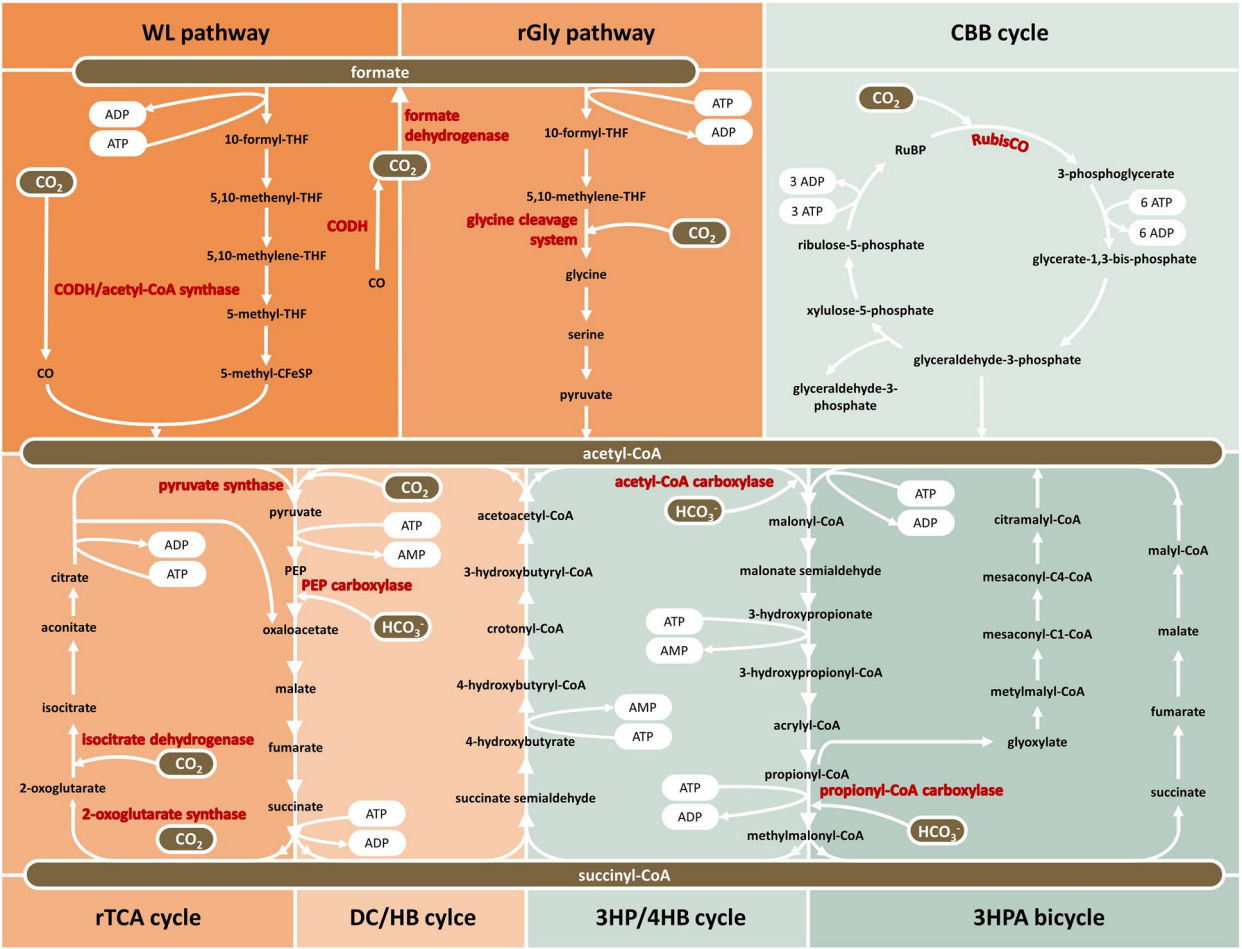
Currently known seven natural autotrophic CO_2_ fixation pathways. The respective carbon fixing enzymes (red) and ATP demand are depicted. Aerobic pathways (mint) include the CBB cycle, 3HP/4 HB cycle and 3HPA bicycle. The WL pathway, rGly pathway, rTCA cycle and DC/HB cycle are anaerobic pathways (orange). (modified after Shi et al. 2020)

CO_2_ assimilation is described as a process of converting CO_2_ into cellular carbon, which requires adenosine triphosphate (ATP) and reducing equivalents. Aerobic microbial organisms require more ATP equivalents, because they use high potential and lower energy electron donors, such as nicotinamide adenine dinucleotide phosphate (NADPH) E0’ ≈ − 320 mV (Berg 2011). In comparison, electron donors with lower potential and higher energy are responsible for providing reducing equivalents in anaerobic microbes. The so far described autotrophic CO_2_ fixing pathways (Fig. 1) have been divided into two groups according to the tolerance of their key enzymes towards oxygen (O_2_). The aerobic pathways include the Calvin Benson Bassham cycle (CBB), the 3-hydroxypropionate bicycle (3HP) and the 3-hydroxypropionate/4-hydroxybutyrate cycle (3HP/4HB), while the reductive tricarboxylic acid cycle (rTCA), the Wood–Ljungdahl pathway (WL), the reductive glycine pathway (rGly) and the dicarboxylate/4-hydroxybutyrate cycle (DC/HB) belong to the anaerobic pathways, since strictly anaerobic enzymes are operating (Berg 2011).

### Calvin Benson Bassham cycle

The CBB cycle is the most important mechanism of autotrophic CO_2_ fixation for common phototrophic microorganisms (Bar-Even et al. 2012) and its key enzyme ribulose-1,5-bisphosphate-carboxylase/-oxygenase (RubisCO) is the most abundant protein in the biosphere, fixing around 10^11^ tons of atmospheric CO_2_ per year (Hayer-Hartl and Hartl 2020). The entire cycle consists of three stages, carboxylation, reduction and regeneration of ribulose-1,5-bisphosphate (RuBP) (Bassham and Calvin 1962). The key enzyme RubisCO catalyzes the carboxylation of CO_2_ and RuBP to generate 3-phosphoglycerate and releasing free energy (ΔrGm′ − 37.8 kJ/mol). 3-phosphoglycerate is subsequently reduced by glycerinaldehyd-3-phosphate (GAP) dehydrogenase and 3-phosphoglycerate kinase to glyceraldehyde-3-phosphate consuming ATP and NADPH (ΔrGm′ =  + 18.7 kJ/mol). Regeneration of 5-bisphosphate takes place through conversion between C3, C4, C5, C6, and C7 sugar, which are finally phosphorylated by phosphoribulokinase to regenerate RuBP (ΔrGm′ =  − 24.2 kJ/mol). One cycle can fix three CO_2_ molecules and produce one GAP molecule at the cost of nine ATP molecules and six molecules of NADPH (Fig. 1). The regeneration of the energy carrier and of reducing equivalents in living microbes is realized by the photosystems. Although the CBB cycle is known to be the most widely used CO_2_ fixation mechanism, the efficiency of carbon assimilation is not very high when comparing it to other naturally occurring pathways. The resulting C3 compound is not suitable for the synthesis of acetyl-CoA, since the conversion of GAP inevitably dissolves CO_2_. However, acetyl-CoA is essential to produce multicarbon compounds, such as fatty acids (Blatti et al. 2013). In addition, large amounts of ATP and NADPH are consumed during this cycle (Berg 2011).

### 3-Hydroxypropionate bicycle

The 3HP bicycle was discovered in photosynthetic green non-sulfur bacteria, i.e., *Chloroflexus* (Mattozzi et al. 2013). In the first cycle, one acetyl-CoA molecule and three bicarbonate (HCO_3_^−^) molecules in total are converted to glyoxylate (ΔrGm′ =  − 109.4 kJ/mol). In the second cycle, acetyl-CoA and pyruvate are generated from glyoxylate trough several steps (ΔrGm′ = − 55.4 kJ/mol). The 3HP bicycle fixes three CO_2_ molecules and produces one pyruvate molecule while consuming seven ATP molecules and five molecules of reducing equivalents (Fig. 1), which makes it more energy demanding than the rTCA and 2HP/4HB cycle (Berg 2011). Although, this cycle is very energy demanding, it is already used in the industry to produce 3HP, which serves as an attractive precursor for acrylate, acrylamide and even as a monomer of biodegradable plastic (Aduhene et al. 2021).

### 3-Hydroxypropionate/4-hydroxybutyrate cycle

The 3HP/4HB cycle has been identified in archaea (Berg et al. 2007). Here, succinyl-CoA is generated from two molecules of HCO_3_^−^ using an acetyl-CoA/propionyl-CoA carboxylase (ΔrGm′ = − 61.9 kJ/mol). The previously generated succinyl-CoA is reduced to 4-hydroxybutyrate, which is then activated to 4-hydroxybutyryl-CoA (ΔrGm′ = − 17.0 kJ/mol), and the key enzyme 4-hydroxybutyryl-CoA dehydratase subsequently synthesizes crotonyl-CoA (ΔrGm′ = − 7.7 kJ/mol). At a final step, crotonyl-CoA is oxidized and cleaved to acetyl CoA (ΔrGm′ = − 16.5 kJ/mol). A full 3HP/4HB cycle uses up two molecules of HCO_3_^−^ to generate one molecule of acetyl-CoA, consuming six ATPs and four reducing NADPH equivalents (Fig. 1) (Berg et al. 2007). Recently, Liu and Jiang improved the activity of the propionyl-CoA carboxylase to enable the efficient synthesis of succinate from acetyl-CoA via the 3HP/4HB cycle (Liu and Jiang 2021), making this autotrophic CO_2_ fixation pathway more attractive for the industry.

### Reductive tricarboxylic acid cycle

The rTCA cycle is found in anaerobic bacteria and photosynthetic green sulfur bacteria (Buchanan and Arnon 1990). This cycle forms acetyl-CoA from two CO_2_ molecules by the consumption of two molecules of ATP (Fig. 1) and reverses the reactions of the oxidative citric acid cycle (TCA) (Berg 2011). For the reversal of the TCA, three rTCA-specific enzymes are required, which include the ATP-citrate lyase, the fumarate reductase as well as the strictly anaerobic ferredoxin-dependent 2-oxoglutarate synthase. Thermodynamically challenging reactions (ΔrGm′ > 10 kJ/mol) of the rTCA cycle are catalyzed by ATP-citrate lyase, 2-ketoglutarate synthase and isocitrate dehydrogenase (Berg 2011). In addition, only recently it was demonstrated that high pressure of CO_2_ can drive the TCA cycle backwards towards autotrophy (Steffens et al. 2021, 2022). This version of the rTCA is identified as the reverse oxidative TCA (roTCA) and mainly differs from the classical rTCA using citrate synthase instead of ATP-citrate lyase, making citrate cleavage thermodynamically challenging (ΔrGm′ > 35 kJ/mol). However, this enables the cell to spend less ATP per acetyl-CoA synthesis from CO_2_ (Mall et al. 2018; Nunoura et al. 2018).

### Dicarboxylate/4-hydroxybutyrate cycle

The DC/HB cycle is also a strictly anaerobic CO_2_ fixation pathway, which converts two molecules of HCO_3_^−^ and acetyl-CoA to succinyl-CoA by a carboxylase–pyruvate-synthase and phosphoenolpyruvate carboxylase. The regeneration of acetyl-CoA is accomplished similarly to the 3HP/4HB cycle. However, the pyruvate synthase and ferredoxin, are inactivated by O_2_. This fixes one molecule of HCO_3_^−^ and one molecule of CO_2_ to generate one molecule of acetyl-CoA at the expense of five ATP molecules (Fig. 1) (Huber et al. 2008; Erb 2011).

### Reductive glycine pathway

In 2020, it was demonstrated that the sulfate-reducing bacterium (SRB) *Desulfovibrio desulfuricans* G11 uses a variation of the linear reductive glycine pathway for carbon assimilation and autotrophic growth (Sanchez-Andrea et al. 2020), confirming the rGly pathway as the seventh natural CO_2_ fixation pathway. Although the main glycine cleavage, is not sensitive to O_2_, autotrophic growth on CO_2_ requires 5,10-methylene tetrahydrofolate (5,10 mTHF). Moreover, the production of 5,10 mTHF costs one molecule of ATP, which is achieved using formate as starting molecule (Fig. 1). The formate is generated by the reduction of one CO_2_ using the oxygen-sensitive formate dehydrogenase (FDH). This first step is shared with the WL pathway (Y. Song et al. 2020), therefore, making aerobic autotrophic growth on CO_2_ using the rGyl pathway not possible. Only recently, Song et al. (2020) were able to confirm the co-utilization of the rGly pathway and the WL pathway under anaerobic autotrophic conditions using ^13^C labeled metabolite tracing and genetic modules. However, among the known CO_2_ fixation routes, rGlyP is also one of the most ATP-efficient pathways, only rivalled by the rTCA cycle and WL pathway (Sanchez-Andrea et al. 2020; Claassens 2021). Therefore, this route could be of industrial interest, but further research will be needed to develop, evaluate and implement potential future applications that base on this recently found CO_2_ fixation pathway.

### Wood–Ljungdahl pathway

In comparison with the main six mentioned CO_2_ fixation pathways above, the WL pathway is characterized to be highly energy efficient as two CO_2_ molecules are fixed to produce acetylCoA by consuming only one ATP (Fig. 1) (Ljungdahl 1994; Hügler and Sievert 2011). This linear exergonic pathway is considered to be the most ancient autotrophic CO_2_ fixation pathway as it is found in both bacteria and archaea (Berg 2011). The WL pathway fixes CO_2_ via a carbonyl (CO) and a methyl (CH3) group using the carbon monoxide dehydrogenase/acetyl-CoA synthase (CODH/ACS) enzyme complex, respectively, to generate acetylCoA (Drake 1994; Fuchs 1994; Ragsdale 2008; Ragsdale and Pierce 2008). The methyl-branch reduces one CO_2_ molecule to formic acid by highly oxygen sensitive FDH (ΔrGm′ =  + 18.0 kJ/mol) and is subsequently attached to tetrahydrofolate to be further reduced. A second CO_2_ molecule is reduced to CO by a nickel atom in the active center of a highly oxygen sensitive CODH as part of the carbonyl-branch. Both reactions are thermodynamically challenging (ΔrGm′ =  + 18.0 kJ/mol and ΔrGm′ =  + 32.6 kJ/mol). Subsequently, the one-carbon unit from the methylbranch is transferred to the nickel bound CO to form actely-CoA (Mock et al. 2015; Jeoung et al. 2019; Lemaire et al. 2020). Unlike the other carbon fixation pathways known so far, CO as an inorganic C1 species is of central importance in the WL pathway. Although toxic to most organisms, CO is necessary for many microorganisms, which have exploited this gas as an energy and carbon source, especially those operating an anaerobic lifestyle (Ragsdale 2004; King and Weber 2007; Jeoung et al. 2014; Robb and Techtmann 2018).

CO is essential for the microbial WL pathway and coupled microbial metabolisms. Moreover, CO is indispensable for a variety of synthetic processes, such as Fischer–Tropsch, Monsanto and Cativa, making it one of the most important C1 feedstocks of the last century (Fujimori and Inoue 2022). Hence, microbes and their natural biocatalysts can be important for industrial processes as they naturally catalyze the required reactions. There are several solutions to seek these microbial biocatalysts from the environment, such as enrichments or cultivations. However, as the vast microbial majority cannot be cultivated to date (Lloyd et al. 2018), an enormous enzymatic potential remains untapped. One way to circumvent the limitation of culture-dependent approaches to identify novel enzymes is functional metagenomics, such as function-based screens (Simon and Daniel 2011, Böhnke and Perner 2015, 2017, Adam and Perner 2018). In the future, such activity-based screens may enable the identification of novel CODHs from the environment with highly valuable properties for industrial application by circumventing the bottleneck of cultivation. An enzyme assay to detect CO oxidation activity of single CODH enzymes using methyl viologen as an electron acceptor already exists (Ensign and Ludden 1991; Seravalli et al. 1995). If such an assay would be upscaled for a functional metagenomic screening, novel CODHs of currently uncultured microbes may be discovered, which may render useful biotechnological applications.

## Reversible reaction between CO_2_ and CO of microbial CO metabolism

Microorganisms that are capable of using CO as an energy source for their growth are mostly referred to as carboxydothrophs (Oelgeschlager and Rother 2008). This includes aerobic and anaerobic microorganisms, which share as a common characteristic the presence of CODH enzymes (Kraut et al. 1989). Nevertheless, CODH enzymes can also be found in other microbes, including carboxydovores, aerobic heterotrophs and acetoclastic organisms (P. S. Adam et al. 2018; Islam et al. 2019). Nevertheless, all CO-oxidizing microorganisms couple the reversible oxidation of CO to the reduction of electron acceptors, which can be either O_2_, protons (H^+^), nitrate (NO_3_^−^) or sulfate (SO_4_^2−^) (King and Weber 2007; Diender et al. 2015; Robb and Techtmann 2018). The reduction of those electron acceptors causes the formation of an ion motive force, which leads to the synthesis of ATP and thus energy production to drive various other metabolic pathways (Meyer and Schlegel 1983). In some cases, CO conversion of SRB though seems to play a role in CO detoxification as it does not result in ATP synthesis and growth in the absence of SO_4_^2−^ (Lupton et al. 1984; Sipma et al. 2006).

### Classification and structure of CODHs

CODHs are classified into two distinct phylogenetic and structurally different groups of aerobic and anaerobic CODHs, primarily based on their sensitivity towards O_2_ (Lindahl 2002; King and Weber 2007; Ragsdale and Pierce 2008; Jeoung et al. 2019). While evolution of anaerobic CODH and CODH/ACS can be defined more easily, evolution of aerobic CODH remains unclear (Weber and King 2010; Diender et al. 2015).

### Aerobic Mo,Cu–CODHs

**Fig. 2:**
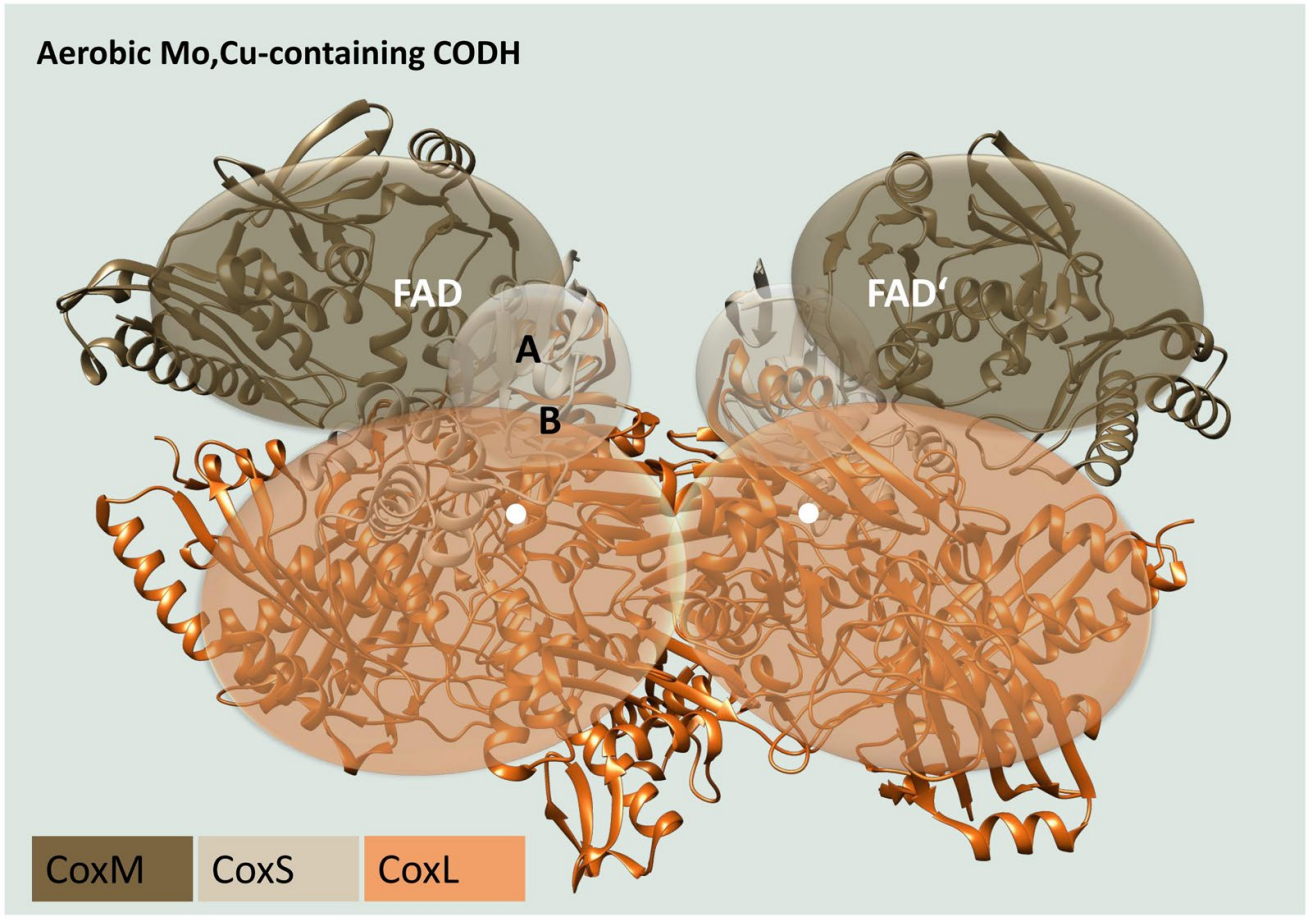
Subunit and cluster composition of aerobic Mo,Cu-containing CODHs. Dimer of heterotrimers, where each heterotrimer is formed by a large subunit containing Mo,Cu active site (CoxL, white dot), a medium FAD containing flavoprotein subunit (CoxM) and a small iron–sulfur subunit (CoxS). FAD, flavo-adenin-dinucleotide; A and B, iron–sulfur–cluster [Fe_2_S_2_]**.** The crystal structure of the Mo/Cu-dependent CODH from *Oligotropha carboxidovorans* in its oxidized form is shown in the background (PDB ID:1N5W) (Dobbek et al. 2002). The graphical design of the crystal structure was performed with UCSF Chimera (Pettersen et al. 2004)

Aerobic CODHs basically differ from anaerobic CODHs in that they are O_2_ tolerant and contain a molybdenum (Mo) metal cofactor, where a copper (Cu) metal binds to a cysteine making it a unique characteristic of aerobic CODHs (Hille et al. 2015; Jeoung et al. 2019). The commonly used designation of aerobic CODHs as Mo,Cu–CODHs was, therefore, obvious (Dobbek et al. 1999; Jeoung et al. 2019). These enzymes belong to the family of molybdenum hydroxylases. Their structure and function have already been intensely studied in the past years (Ragsdale and Kumar 1996; Dobbek et al. 1999; Ragsdale 2004; Jeoung et al. 2014; Hille et al. 2015). Members of this Mo,Cu–CODH enzyme family have two active sites, two [Fe_2_S_2_]-clusters and a flavin adenine dinucleotide (FAD) functioning as an electron acceptor (Fig. 2) (Jeoung et al. 2014). The Mo,Cu–CODH consists of three subunits (CoxS, M, L), that are encoded in a single gene cluster (Resch et al. 2005). The large subunit contains a molybdenum cysteine dinucleotide that places the catalytically essential molybdenum atom at the active site of the enzyme and is responsible for CO hydroxylation (Meyer et al. 2000; Jeoung et al. 2014). The medium subunit orientates the FAD cofactor, while the small subunit carries two [Fe_2_S_2_]-clusters. Altogether, a dimer consisting of heterotrimers is formed in a butterfly shape (Fig. 2) (Jeoung et al. 2014). To date, two different forms of aerobic CODHs have been described. The first form (EC 1.2.5.3) uses quinones as electron acceptors (Wilcoxen et al. 2011), while form II (EC 1.2.2.4) is described as taking advantage of cytochrome b as an electron acceptor (Meyer et al. 1986). However, the aerobic form II of this CODH is still under discussion and, therefore, remains a putative CODH (Xavier et al. 2018).

#### Anaerobic Ni,Fe–CODHs (EC 1.2.7.4)

**Fig. 3:**
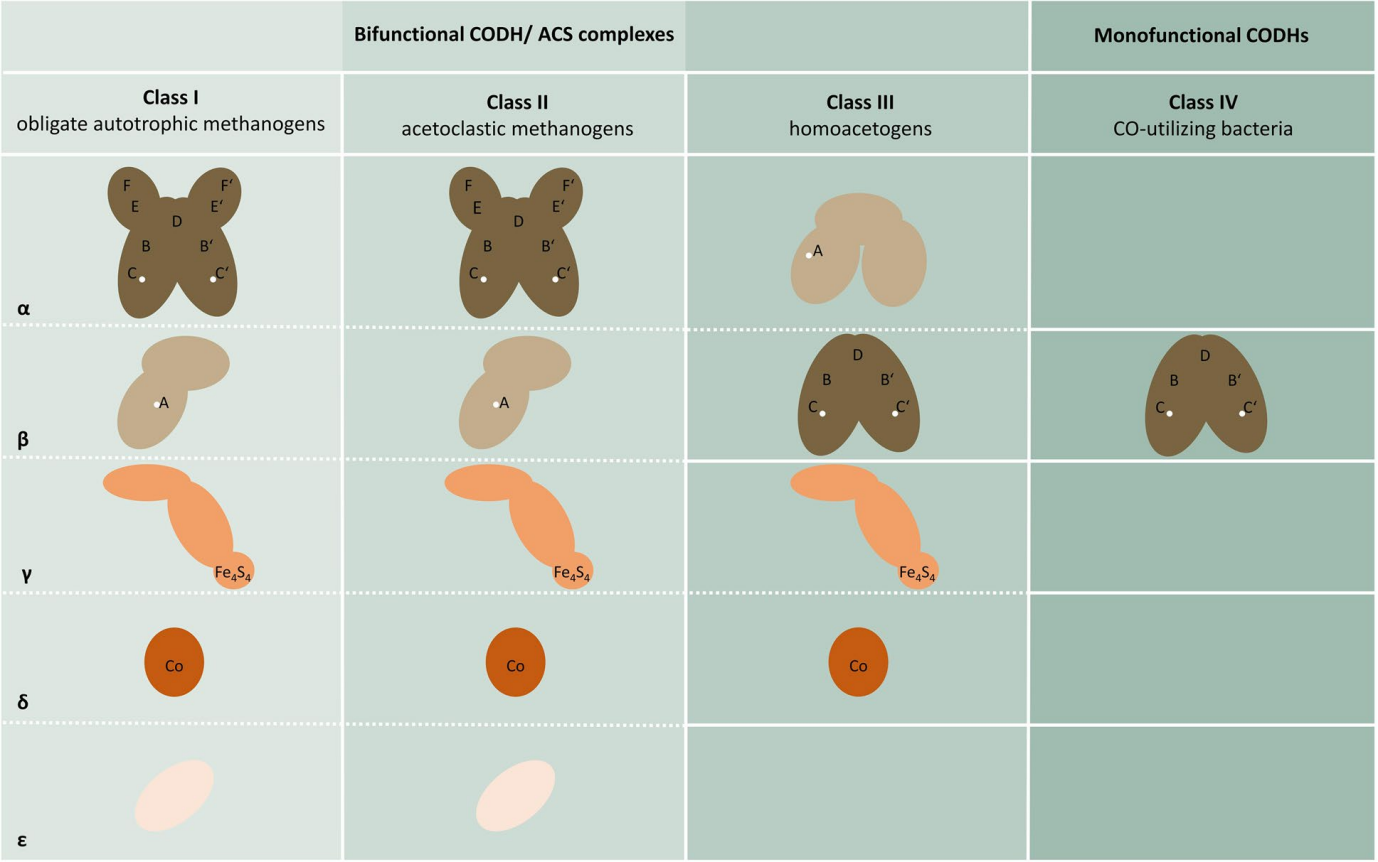
Subunit and cluster composition among the four classes of Ni,Fe-containing CODHs. Homologues proteins are illustrated by the same shade of brown color. Different iron–sulfur clusters are visualized by capital letters, while subunits are presented by Latin characters. Active sites containing a nickel center are shown by a white dot. Protein complexes formed by corresponding subunits are indicated by dashed lines (modified after Jeoung et al. 2019).

In comparison with aerobic Mo,Cu–CODH enzymes, anaerobic CODHs possess mostly an active Ni,Fe-center, which makes them highly sensitive towards O_2_ (Merrouch et al. 2015; Jeoung et al. 2019; Biester et al. 2022). They are referred to as Ni,Fe–CODHs. Most anaerobic CODHs contain nickel and iron which are part of a cofactor for binding CO at the active site. Studies on the activation at the Ni,Fe-cluster state that enzyme’s active center within the C-cluster feature a hydroxyl group bound to an asymmetrically coordinated Fe ion close to the Ni. During the binding of CO to the Ni–metal center, a change in the geometry occurs, which is caused by the nucleophilic attack of the hydroxide on the carbonyl carbon. This results in the formation of an Ni–C(O)O–Fe intermediate, which subsequently decomposes due to the release of CO_2_. This implies that the C-cluster harbors an Ni-bound hybrid that is released as a proton by the loss of electrons (Volbeda and Fontecilla-Camps 2005; Jeoung and Dobbek 2007; Boer et al. 2014). These Ni,Fe–CODHs feature a variety of different subunit compositions, differing in size and their physiological functions and are thus, divided into four classes (Fig. 3) (Lindahl 2002).

Class I and II CODHs are only found in archaea, especially in methanogens (Jeoung et al. 2019). They consist of five different subunits, forming an oligomeric complex of which only the alpha-subunit owns the CODH enzymatic activity, while the beta-subunit harbors the active site nickel–iron–sulfur cluster of the acetyl-CoA synthase (Fig. 3) (Grahame and DeMoll 1995) Class III CODH enzymes are found in strictly anaerobic bacteria and archaea, predominantly in acetogenic bacteria (Jeoung et al. 2014, 2019). This class of CODHs are described as bifunctional CODH/ACS, which is a five-domain containing enzyme complex. It has the additional function of cleaving acetyl-CoA into a methyl group, coenzyme A, and CO, which is not the case for monofunctional CODHs. This reaction is reversible, with CODH/ACS forming acetyl-CoA (Ragsdale and Kumar 1996, Doukov et al. 2002, Ragsdale 2004, Adam et al. 2018). Grahame et al. (2005) figured out that the ACS reaction seems to be freely reversible and, therefore, is not forcing any direction of the reaction. Although bacterial CODH and ACS are connected via a hydrophobic tunnel, both enzymes can also be found independently from each other, which reflects their bifunctionality. Moreover, this gas channel protects the cell against the toxicity of CO, as carbon source cannot escape into the environment but is sequestered by microbes for metabolic reactions (Seravalli and Ragsdale 2000; Svetlitchnyi et al. 2001; Lindahl 2002). Nevertheless, bifunctional CODH/ACS and corrinoid iron–sulfur protein (CFeSP) are encoded in operons forming a functional unit. Class IV anaerobic CODHs are so called monofunctional CODHs, as these enzymes catalyze the reversible conversion of CO to CO_2_ only, using CO mainly as an electron source, like in *Rhodospirillum rubrum* and *Carboxydothermus hydrogenoformans* (Drennan et al. 2001; Wu et al. 2005; Alfano and Cavazza 2018). Although they lack the ACS, most of the structures, such as the active site and the arrangement of the [Fe_4_S_4_] cluster, as well as the activation of CO_2_, are homologous to bifunctional CODHs class III (Fig. 3) (Lindahl 2002).

### Distribution of CODHs

CODHs are very ancient enzymes as they are present in phylogenetically and physiologically diverse bacteria and archaea (Martin and Russell 2007; Jeoung et al. 2014). Interestingly, Techtmann et al. (2012) calculated that about 6% of all known microbial genomes consist of at least one Ni,Fe–CODH encoding gene, suggestive for anaerobic CO-utilization being widespread through the microbial world. The increasing number of newly discovered bacterial and archaeal genomes encoding genes for the catalytic subunit of CODHs indicates that microbes from geographically and chemically distinct environments (Hoshino and Inagaki 2017; Inoue et al. 2018, 2022; Peng et al. 2021) may use CO oxidation as their main carbon source or as a backup energy source (King and Weber 2007; Techtmann et al. 2012). Consequently, it is highly likely that among the uncultured microbial majority (81% of microbial cells on earth) numerous, currently inaccessible CODH (-like) enzymes are hidden (Lloyd et al. 2018). Targeting, identifying and characterizing this tremendous potential of CODH (-like) biocatalysts must be one of the key strategies used in future research approaches (Böhnke and Perner 2022).

## CODH-coupled metabolisms

**Fig. 4:**
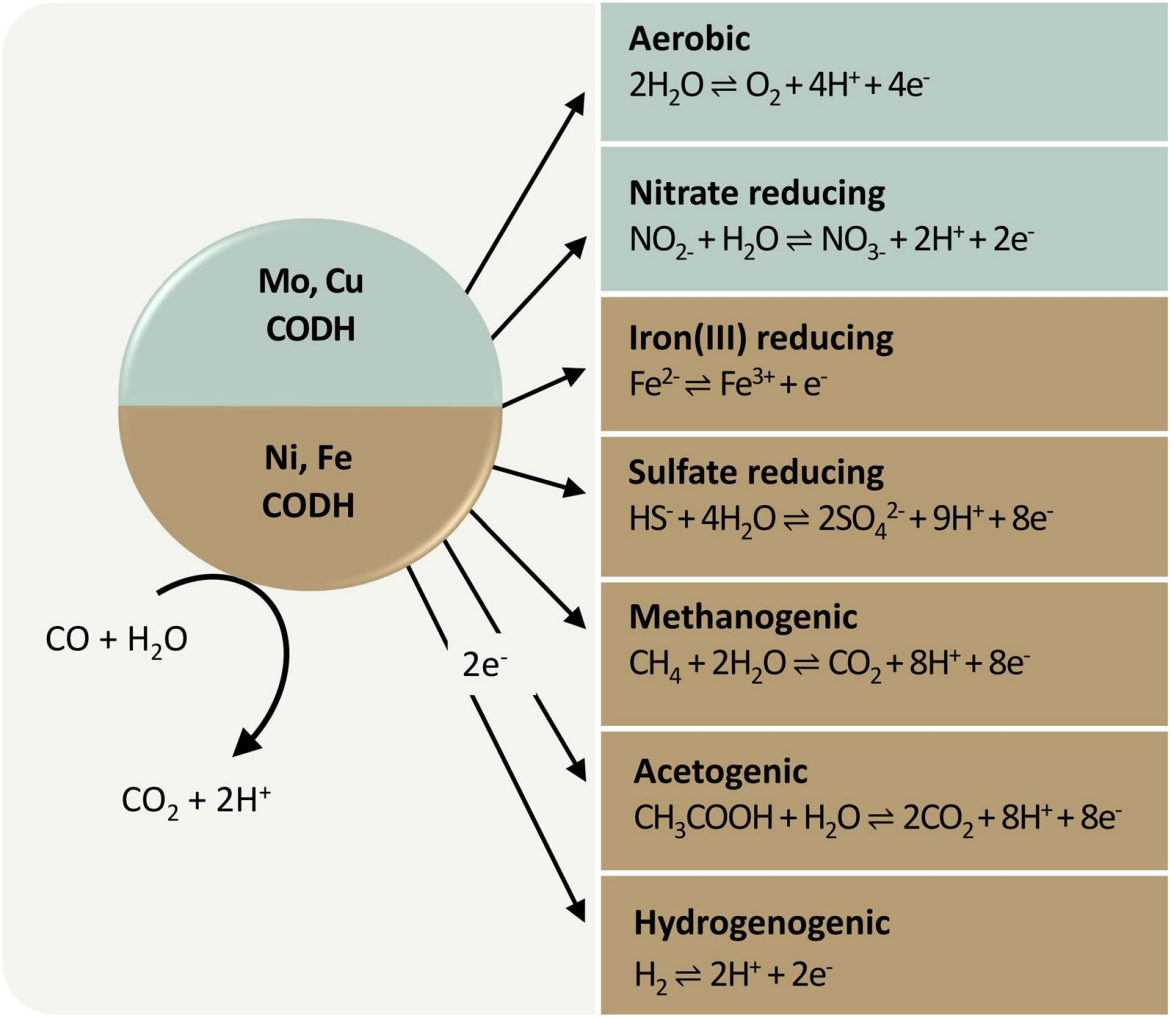
CO oxidation-coupled energy conservation metabolisms by aerobic Mo,Cu–CODHs (mint) and anaerobic Ni,Fe–CODHs (brown)

Kluyver and Schnellen’s lab was the first to observe microbial CO oxidation (Kluyver and Schnellen 1947). Since their observation, CO metabolisms moved into a scientific focus. This is due to the fact that CO is an important intermediate compound not only in the aerobic, but also in the anaerobic carbon cycle. CO is also capable of fueling various metabolic processes, such as acetogenesis, methanogenesis, hydrogenogenesis, and aerobic carboxydotrophy (Fig. 4) (Pugh and Umbreit 1966; Ragsdale and Pierce 2008; Diender et al. 2015; Jones et al. 2016; Robb and Techtmann 2018).

### Aerobic CO metabolism

Energy conservation from CO of carboxydotrophs is used to synthesize biomass from CO_2_ via autotrophic carbon fixation, which involves the CBB cycle and ATP generation through the aerobic respiratory chain (Xavier et al. 2018). One well-studied representative organism that is able to couple CO metabolisms to the CBB cycle is the alphaproteobacterial carboxydotroph *Oligothropha carboxidovorans* (Mörsdorf et al. 1992; Siebert et al. 2022). This aerobic growth on CO as sole energy and carbon source has also been found in Actinobacteria, Bacilli and Gammaproteobacteria (Zavarzin and Nozhevnikova 1977; Krüger and Meyer 1984; Anand and Satyanarayana 2012). A second microbial group named carboxydovors, which includes the Mycobacteria (King 2003b, a) are also able to oxidize CO aerobically. However, it is assumed that carboxydovors support aerobic respiration without being linked to carbon fixation from CO (Cordero et al. 2019). However, regardless of whether carboxydotrophs or carboxydovores are involved, the aerobic respiration driven by CO oxidation always proceeds according to Eq. (1) and is catalyzed by either of the two different aerobic CODH enzymes, namely, a membrane-bound CODH and a cytoplasmic CODH:

1 $$2 \, CO \, + \, O_{2} \to 2 \, CO_{2}\, \Delta G^{\prime}_{0} = \, {-}514 \, kJ \, / \, mol \, CO.$$

The membrane-bound CODH generates energy through the oxidation of CO with water to CO_2_. Electrons and protons that are provided by this reaction are transferred to the CO-intensive respiratory chain. Subsequently, these are accepted by a cytochrome b complex or a quinone, which can then either lead to O_2_ reduction (Jacobitz and Meyer 1989) or NO_3_^−^ reduction (Frunzke and Meyer 1990; King 2006). The motive force resulting from this process is then used to generate ATP. The second CODH, located in the cytoplasm, is involved in hydrogen (H_2_) evolution (Mörsdorf et al. 1992). CO_2_ that is generated by CO oxidation is then assimilated within the CBB cycle via the RubisCO to support CO_2_ fixation (Meyer and Schlegel 1983; King and Weber 2007; Xavier et al. 2018).

### Anaerobic CO-coupled metabolisms

#### Acetogenesis and the Wood–Ljungdahl pathway

Acetogens are obligate anaerobic bacteria that are able to fix CO_2_ into acetate via the linear, two branched reductive acetyl-CoA pathway, well-known WL pathway (Lynd et al. 1982, Ljungdahl 1994, H. L. Drake et al. 2002). They use the WL pathway not only for the fixation of CO_2_ according to Eq. 2, but also for redox balancing. Over the last century, this autotrophic carbon fixation pathway has been excessively investigated in acetogens. However, studies conducted with non-acetogens have shown that some representatives are also capable of assimilating CO_2_ via this route (Diekert and Thauer 1978; Ragsdale and Pierce 2008; Robb and Techtmann 2018):

2 $$\begin{aligned}&4CO \, + \, 2H_{2} O \rightleftharpoons CH_{3} COO^{-} + \, H^{ + } + 2CO_{2}\\ & \Delta G^{\prime}_{0} = \, {-} \, 43.6\text{kJ} \, / \, \text{mol} \, \text{CO}.\end{aligned}$$

As already mentioned, the WL pathway consists of an eastern (methyl-) and a western (carbonyl-) branch in which two molecules of CO_2_ are reduced (Ragsdale 2008). The eastern branch provides a methyl group, which is generated by the energetic reduction of one molecule of CO_2_. The heteroatoms to which the methyl group is attached are protonated, in order for it to be electrophilically activated and transferred towards CFeSP (Ragsdale 2008). CFeSP bound to an acetyl-CoA-synthesis complex allows the methyl group to be supplied for subsequent condensation (Ragsdale 2008; Ragsdale and Pierce 2008). Reduction of the second CO_2_ to CO within the western branch is performed by the CODH. The CODH/ACS synthase complex then finally catalyzes the condensation of the methyl residue, the carbonyl residue, and coenzyme A to acetyl-CoA, which is further converted to acetate (Drake 1994; Ragsdale and Pierce 2008). It has been demonstrated, that CO is also metabolized by acetogens via the WL pathway coupling acetogenesis to the formation of an ion motive force, which results in ATP synthesis (Diekert and Thauer 1978; Müller 2003). Moreover, several steps of CO_2_ fixation in the WL pathway require input of electrons, wherefore different types of cofactors are needed. These steps differ for each microorganism and enzyme, which makes a predication of a general acetogenic CO metabolism almost impossible (Sim et al. 2007; Hess et al. 2013).

Two of the most studied acetogenic bacteria are *Moorella thermoacetica* (homoacetogen) and *Acetobacterium woodii*, both showing different approaches of acetogenesis (Müller et al. 2008; Hess et al. 2013; Bertsch and Müller 2015). *A. woodii* oxidizes CO by its CODH, whereby ferredoxin is reduced. An RnF complex (energy-converting NADH:Fdox oxidoreductase) links the following (re-) oxidation of ferredoxin to the reduction of NAD^+^. This process results in a transmembrane Na^+^ translocation, which forces ATP generation (Biegel and Müller 2010; Biegel et al. 2011). NADH and reduced ferredoxin can then additionally be used to generate molecular H_2_ by an electron-bifurcating hydrogenase. Moreover, a H_2_-dependent CO_2_ reductase is postulated to use the reduced ferredoxin as an alternative electron donor for the CO_2_ reduction to acetate (Schwarz et al. 2020). However, acetogens using RnF complexes have to couple the CO-oxidation to the WL pathway as they cannot couple oxidation of ferredoxin to the reduction of proton directly (Diender et al. 2015). In contrast, *M. thermoacetica* differs from *A. woodii* in that these acetogens do not contain RnF complexes but instead harbor energy-converting-translocating hydrogenases (EcHs).

### CO-coupled hydrogenogenic metabolism

Although the ancient reductive acetyl-CoA pathway has been employed by acetogens to form acetate, an additional mechanism for ATP generation is needed for chemolithoautotrophic growth as the central pathway does not supply ATP via substrate-level phosphorylation (Diender et al. 2015). Schoelmerich and Müller (2019) recently demonstrated that EcH functions as a respiratory enzyme, which establishes a chemiosmotic gradient. Their experiments reveal that CO oxidation can indeed be coupled to H_2_ production and the formation of transmembrane electrochemical ion gradients. In more detail, hydrogenogenic oxidation of CO is commonly known as water–gas-shift reaction (see Eq. 3) and results in the generation of H_2_ and CO_2_. Enzymes involved in this reaction include Ni,Fe–CODH, electron transfer proteins, and EcHs. The electrons gained from the CODH catalyzed CO oxidation are transferred via a ferredoxin-like carrier, which is subsequently oxidized coupled to proton reduction using an EcH complex (Fukuyama et al. 2020). This reaction does not only lead to the formation of a proton motor force, but also to the release of H_2_ (Hedderich and Forzi 2005). In the past, numerous hydrogenogenic CO metabolizing microbes have been investigated, with a focus on *M. thermoacetica, R. rubrum*, *C. hydrogenoformans* and *Thermoanaerobacter kivui* (Kerby et al. 1992; Huang et al. 2000; Svetlitchnyi et al. 2001; Diender et al. 2015; Schoelmerich and Müller 2019).

3 $$\begin{aligned} CO \, + \, H_{2} O \rightleftharpoons CO_{2} + \, H_{2} \\ & \Delta G^{\prime}_{0} = \, {-}20kJ \, / \, mol CO\end{aligned}$$

In *R. rubrum*, there are two operons encoding the associated enzyme complex known as Coo. The cooF–SCTJ operon encodes the CODH and related proteins, and the cooMKLXU operon encodes a CO-induced hydrogenase (Fox et al. 1996a, 1996b). Heme-protein (CooA) is found to function as a CO sensor and, therefore, controlling the transcription of the enzymatic machinery needed for chemoautotrophic growth (Roberts et al. 2001). Electrons provided by CO oxidation are shuttled through an iron–sulfur protein (CooF), which is directly associated with the CODH, to the EcH. Not only does the CODH of *R. rubrum* catalyze the reaction of CO to CO_2_ very efficiently but additionally, CO-induced hydrogenase of *R. rubrum* is highly CO tolerant and, therefore, well-adapted to growth on CO (Bonam et al. 1984; Fox et al. 1996b; Singer et al. 2006). *C. hydrogenoformans* is so far the best-known microorganism having multiple CODHs encoding genes on its genome (Wu et al. 2005). Although the metabolism was initially described as strictly fermentative, later studies by Henstra and Stams demonstrated additional growth by respiration on CO (Henstra and Stams 2004). Increasing H_2_, CO_2_ and acetate concentrations driven from CO oxidation could also indicate that the WL pathway acts as backup for the hydrogenogenic metabolism of *C. hydrogenoformans* (Henstra and Stams 2011).

### CO-coupled methanogenic metabolism

Besides acetogens, methanogens are able to grow with CO as their sole energy source. The majority of methanogens, e.g., *Methanococcus maripaludis* reduces CO_2_ to methane (CH_4_) and uses H_2_ as electron donor. In this case CO_2_ can either be used directly or be generated by CO oxidation via a membrane-bound monofunctional CODH in the first step (Ferry 1999; Oelgeschlager and Rother 2008). CO_2_ can then be converted into formyl-methanofuran to enter the pathway for CH_4_ production. In addition, CO_2_ can be used for carbon assimilation directly by bifunctional CODH/ACS complexes or coming from methylene–tetrahydromethanopterin (Nagoya et al. 2021). These reactions are fueled by electrons, which are generated via H_2_ oxidation. This H_2_ oxidation can be carried out by various hydrogenases, including membrane-bound EcH, F_420_-non-reducing hydrogenases, cytoplasmatic F_420_-reducing hydrogenases as well as cytochrome-b-containing heterodisulfide reductases (Schöne and Rother 2018; Nagoya et al. 2021). Finally, ATP is generated by either a H^+^ or Na^+^ translocating ATPase, where Na^+^ is provided by the membrane-bound methyl-H4MPT:coenzyme M methyltransferase. However, CO utilization with methanogenesis according to Eq. 4 is relatively inefficient, which is reflected by a ΔG’_0_ of –52.6 kJ/mol CO, resulting in slow growth rates (O'Brien et al. 1984). This might be caused by the toxic nature of CO as well as that CO-metabolism moves easier towards CH_4_ alternative products (Schöne and Rother 2018):

4 $$4CO \, + \, 2H_{2} O \to CH_{4} + \, 3CO_{2}^{ } \,\,\Delta G^{\prime}_{0} = \, {-} \, 52.6 \, kJ/mol \, CO.$$

Other methanogens such as *Methanosarcina* species couple the WL pathway to acetolactic methanogenesis (Thauer 1988; Ferry 1999; Oelgeschlager and Rother 2008). This process is also described as fermentation, since acetate is cleaved and methyl groups are reduced to methane with electrons derived from the oxidation of the carbonyl group to CO_2_. The cleavage of the activated acetate is performed by phosphotransacetylase and acetate kinase, while a bifunctional CODH/ACS complex (Lyu et al. 2018; Nagoya et al. 2021) subsequently converts acetyl-CoA into CO, methyl-group and coenzyme A. Later, CODH/ACS then oxidizes this CO to CO_2_. Electrons provided by the reaction are accepted and transported by ferredoxin to reduce the methyl-group to CH_4_ according to the reactions of hydrogenogenic methanogenesis (Fischer and Thauer 1990; Schöne and Rother 2018). Most acetoclastic methanogens use EcH and F_420_-non-reducing hydrogenase to reoxidize ferredoxin. This mechanism is similar to the H_2_ oxidation of hydrogenogenic methanogens. In contrast, some acetoclastic methanogens have evolved RnF to drive the ion motive force as they lack both EcH and F_420_-non-reducing hydrogenase (Ferry 2010). However, this process usually results in degradation of biomass, as they rely on acetate degradation (Schöne and Rother 2018; Nagoya et al. 2021).

### Sulfate reduction coupled to CO oxidation

Most SRB have shown low tolerance towards CO and it has even been reported to be toxic to them. Therefore, CODHs have been mostly considered to function in CO detoxification mechanisms (Parshina et al. 2005a; Matsumoto et al. 2011; Alves et al. 2020). When growing on pyruvate, cleavage of this substrate results in the production of 2 acetyl-CoA, 2 H_2_O and 2 CO (Voordouw 2002; Sipma et al. 2006; Diender et al. 2015). Toxic CO is then funneled and converted into 2 CO_2_ and H_2_ via a monofunctional CODH and membrane bound CO-dependent hydrogenase. Subsequently a periplasmatic hydrogenase generates H^+^ and electrons, which are transported via a cytochrome c network to a transmembrane electron transport complex (e.g., Hmc). The formation of acetate additionally provides ATP, which is later used for SO_4_^2−^ reduction by SO_4_^2−^ reducing enzymes (e.g., ATP sulfurylase) using the generated protons and electrons (Voordouw 2002; Diender et al. 2015). Several studies on CO metabolism of SRB have shown growth on organic electron donors, such as lactate and pyruvate, resulting in acetate production, to be most likely:

5 $$4CO \, + \, SO_{4}^{2 - } + \, 4H_{2} O^{ - } \rightleftharpoons 4HCO_{3}^{ - } + \, HS^{ - } + \, 3H^{ + } \quad \Delta G^{\prime}_{0} = \, - 37.1 \, kJ/mol \, CO.$$

However, exceptions such as *Desulfovibrio vulgaris* strain Madison exit. This SRB was the first demonstrated coupling direct CO oxidation to SO_4_^2−^ reduction, generating CO_2_, H_2_, and H_2_S as end products when cultured in the presence of SO_4_^2−^ according to Eq. 5. The generated H_2_ is subsequently used for SO_4_^2−^ reduction (Lupton et al. 1984; Rabus et al. 2006). This leads to the hypothesis that CO can indeed be a direct electron donor for thermophilic (Hocking et al. 2015) and mesophilic carboxydothrophic SRB (Parshina et al. 2010). It is assumend though that thermophilic microbes tolerate the presence of CO better (Parshina et al. 2005a). Moreover, *Desulfotomaculum carboxydivorans* strain CO-1-SRB was demonstrated to grow under 100% CO atmosphere using CO as an external electron donor for SO_4_^2−^ reduction. No SRB has previously been reported tolerating such high concentrations of CO (Parshina et al. 2005b). This opens space for further discussions of SRB being a potential source to drive biological SO_4_^2−^ reduction using CO as electron donor, especially when co-cultured (Sinharoy et al. 2020).

## Electrochemical applications of CODH enzymes

### Principles and electrochemical mechanisms

In stark contrast to biological CO oxidation and CO_2_ reduction occurring readily at or near room temperature, the chemical activation of the linear molecule CO_2_ is challenging, since it usually involves a thermodynamically unfavorable one-electron reduction step (Appel et al. 2013; Schlager et al. 2017a):

6 $$CO_{2} + \, e^{-} \to CO_{2}^{{ \bullet {-}}} \quad E_{0} = \, {-}1.9 V \, at \, pH 7.$$

**Fig. 5:**
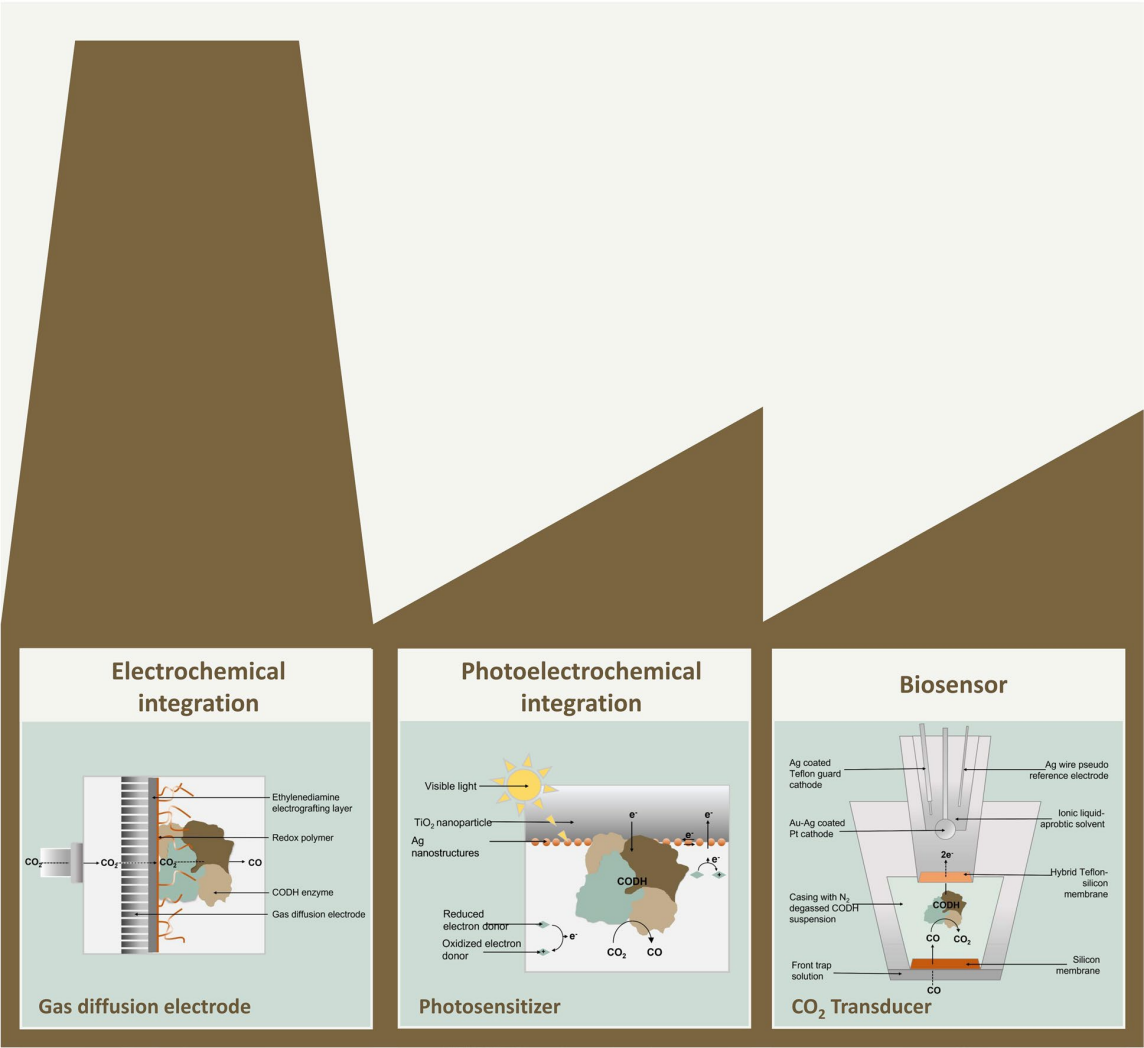
Electrochemical applications of CODH enzymes in industry.

CODH enzymes circumvent this energetically adverse step by allowing for a direct two-electron proton-coupled electron transfer towards CO (Fesseler et al. 2015; Ribbe 2015; Sultana et al. 2016). Due to this inherent property of catalyzing the interconversion between CO_2_ and CO reversibly with little overpotential, CODH enzymes have been used in several different applications, e.g., as biosensors for CO detection or as catalysts for biosynthesis applications (Fig. 5). These utilizations can be achieved using either live microbes as cultures or the purified enzyme only (Shin et al. 2003, Song et al. 2011). To this end, CODH from both anaerobic and aerobic sources have been used, albeit typically towards distinct applications. While the anaerobic Ni,Fe–CODH enzymes perform reversibly and can thus be exploited for biosynthesis via CO_2_ reduction, their aerobic Mo,Cu-based counterparts are strictly limited to CO oxidation and are, therefore, limited to gas sensing applications (Reginald et al. 2019; Contaldo et al. 2021; White et al. 2022).

If only the CO_2_ reduction half-reaction (CO_2_RR) or its inverse, the CO oxidation, is performed in the absence of a complementary half-reaction, then electrons must be provided from an electrode or drawn to it: this means that the enzyme is used in an electrocatalytic system. The main challenges for the utilization of CODH enzymes in electrocatalysis are their immobilization on the electrode surface and their stability related to either leaching or a low tolerance towards O_2_ (Alfano and Cavazza 2018; Reginald et al. 2022). The electronic communication pathway generally considered as the most favorable between enzyme and electrode is the direct electron transfer (DET) via immediate contact of the biomolecule to the solid surface. This configuration enables fast electron transfer and ensures that the electrical potential experienced at the active site is equal to that applied by the external potentiostat (Reginald et al. 2022). This configuration is challenging to achieve, since the electron tunneling efficiency is strongly dependent on the distance between the electrode and the enzyme’s redox cofactors and the enzyme’s geometric orientation on the electrode is difficult to control (Page et al. 1999; Freire et al. 2003). To this end, strategies such as the employment of linkers can help minimize this distance and, therefore, support DET (Woolerton et al. 2011; Contaldo et al. 2022; Reginald et al. 2022). In a simpler approach, enzymes are immobilized on carbon-based electrodes by co-adsorption with polymyxin (Hoeben et al. 2008). The resulting non-specific interactions of CODHs and electrode through physical adsorption have shown to be sufficient to enable DET (Wang et al. 2013a, 2013b). Alternatively, enzymes can be immobilized at a longer, and less accurately defined, distance from the electrode surface. In this case, then, electron transfer can be supported by redox mediators with favorable negative redox potential values. This approach is known as a mediated electron transfer (MET). To mediate the bioelectrochemical reduction of CO_2_, small molecules such as viologens or diquats can be used as reducing agents for the enzyme (Shin et al. 2003; Amao and Ikeyama 2015; Ikeyama and Amao 2016; White et al. 2022). Fundamentally, they artificially replace mediator compounds, such as ferredoxins or NADH, which serve this purpose in vivo (Bender and Ragsdale 2011; Amao and Ikeyama 2015). In this case of a mediated electron transfer, immobilization of CODHs can be achieved by their entrapment close to the electrode within a polymer redox hydrogel (Becker et al. 2022). Other commonly used enzyme immobilization strategies to be combined with mediated electron transfer include the cross-linking of proteins by employing bifunctional agents, such as glutaraldehyde or the immobilization of enzymes within a sol–gel (David et al. 2011; Datta et al. 2013). Both approaches have not yet been reported for CODHs.

### Practical electrochemical implementation

Fundamental investigation of CODH electrochemistry and electrocatalytic reaction mechanisms must rely on DET occurring at the surface of perfectly planar electrodes. In this so-called protein film electrochemistry (PFE) configuration, enzymes are bound directly to the working electrode and can be studied in the best-controlled conditions possible: the dependence of turnover (quantified as electrical current density) when varying the applied potential, the substrate-to-product ratio, the concentration of possible inhibitors, the pH, or further experimental parameters, provides crucial indirect evidence pertaining to the individual chemical reaction steps while requiring only minute amounts of enzyme to perform the analysis (Léger et al. 2003; Parkin et al. 2007; Wang et al. 2013a, 2014, 2013b).

Let us now consider some prominent cases of electroenzymatic CO_2_ to CO conversion with CODHs. The first report was by Shin et al. in 2003, who utilized CODH from *M. thermoacetica* and demonstrated turnover frequencies (TOF) of 700 h^–1^ at less than 100 mV applied overpotential (Shin et al. 2003). Recently, efforts have been made to integrate CODH on gas-diffusion electrodes towards the CO_2_RR to avoid possible mass transport limitations. Contaldo et al*.* used monofunctional CODH from *R. rubrum* on gas-diffusion electrodes, catalyzing the reversible CO_2_/CO interconversion with turnover frequencies up to 150 s^–1^ for CO oxidation at 250 mV overpotential and 420 s^–1^ for CO_2_ reduction at 180 mV overpotential while reaching a device stability of several hours (Contaldo et al. 2021). Becker et al*.* used a cobaltocene-based redox polymer to immobilize CODH II from *C. hydrogenoformans* on gas diffusion electrodes (Fig. 5) and simultaneously serve as the redox mediator, reporting CO_2_RR current densities up to – 5.5 mA cm^–2^ at an applied potential of – 0.79 V vs. SHE (standard hydrogen electrode). This corresponds to a TOF of 2.7 s^–1^ at about 150 mV overpotential. The electrodes showed improved stability with a performance half-life of more than 20 h (Becker et al. 2022).

Further electrocatalytic applications of CODHs aim at generating a product different from CO, and, thus, couple the CODH-catalyzed step with a subsequent or complementary reaction. For example, CODHs have been electronically coupled with hydrogenases (enzymes converting H_2_ ⇌ 2H^+^  + 2e^−^) by immobilization on electrically conductive graphite platelets (Lazarus et al. 2009). This allows one to perform two complementary electrochemical half-reactions while omitting the use of an external circuit, since by catalyzing the oxidation of CO, electrons are directly supplied to the hydrogenase and used towards the reduction of protons and, therefore, hydrogen evolution. This provides a biological alternative to the industrially important water–gas shift reaction, which usually requires higher temperatures and harsher overall conditions (Lazarus et al. 2009). CODH can also be utilized when still in vivo, using CODH-containing microbes towards the electrochemical CO_2_ reduction. In this case, it is essential to use a mediator for electron transfer, because the cell walls prevent DET. The selectivity towards CO as reaction product is decreased due to the presence of other enzymes, including FDH (Song et al. 2011).

### Photoelectrochemical integration

In a further step of integration, CODH enzymes have also been employed as catalysts in the photoreduction of CO_2_ to CO that is, the direct use of sunlight energy to generate electrons and reduce CO_2_. This was achieved by coupling the enzyme to a light-harvesting component, such as semiconductor nanostructures with suitable bandgaps or dyes, providing “hot” electrons for catalytic turnover after excitation. The electrons needed to regenerate the dye or semiconductor after photoinjection of charge carriers can originate either from a sacrificial electron donor or from performing water oxidation separately in a second half-reaction (Woolerton et al. 2012). Woolerton et al*.* immobilized CODH I from *C. hydrogenoformans* on TiO_2_ nanoparticles together with a ruthenium bipyridyl photosensitizer and reported a TOF of 0.14 s^–1^ using visible light irradiation (Woolerton et al. 2010). Coupling CODH to CdS nanorods instead improved the average TOF (per CODH) to 1.23 s^–1^ (Chaudhary et al. 2012). Co-immobilization of a CODH I together with Ag nanoclusters on TiO_2_ nanoparticles (Fig. 5) constitutes the most efficient CODH-based photoreduction installment up to date, with a reported TOF of 20 s^–1^ at room temperature under visible light irradiation (Zhang et al. 2018). Recently, also CODH II from *C. hydrogenoformans* was used as a CO_2_RR catalyst on a light-absorbing CdSe/CdS heterostructure with TOF of 9 s^–1^ and quantum yields up to 19% (White et al. 2022). The enzymes’ TOF in all photoreduction applications is always significantly lower than their inherent activities, which is attributed to a combination of distinct factors: absorption of photons and delivery of charge carriers, recombination of carriers, electron transfer issues, CODH leaching, or enzyme deactivation by O_2_ (Woolerton et al. 2010, 2012; White et al. 2022).

### Biosensors

The use of CODH in a CO biosensor is usually also based on the establishment of electronical communication between the enzymes catalyzing CO oxidation and a working electrode and the subsequent analysis of the amperometric response when exposed to the CO analyte. The first functional CODH-based CO sensor was reported by Turner et al. (Turner et al. 1984), where the purified enzyme from *Pseudomonas thermocarboxydovorans* was coupled to an Au electrode via cytochrome C, allowing for the quantification of CO in both aqueous and gaseous media. Recently, sensing of CO in solution was achieved by utilization of a DET-capable oxygen-tolerant Mo,Cu–CODH from *Hydrogenophaga pseudoflava*, immobilized on an Au electrode without the need for any mediator (Reginald et al. 2019). The same group then simplified the system to a recombinant CODH subunit from the same biological source to build a Clark-type CO bio-microsensor (Fig. 5) capable of detecting CO concentrations from 15 nM to 0.9 µM. The device retains approximately 80% activity and selectivity after 1 week of continuous operation (Reginald et al. 2021).

## Conclusions

The earth’s atmosphere contains several hundred gigatons of CO_2_ and high CO_2_ levels in exhaust chimneys of industrial processing are emitting on a daily basis into the atmosphere. During the past few decades, intensive research on the central carbon-metabolizing enzymes of the autotrophic CO_2_ fixation pathways has been conducted to capture carbon efficiently and cleanly through enzymatic biocatalysts. Comparing all known natural CO_2_ fixation pathways, the WL pathway is the most energy efficient by consuming only one ATP. In this respect, its enzymes are of great interest. In particular CODHs, since they act in a variety of metabolic pathways and can be used for synthesis of sustainable substances, such as acetate or isopropanol. In addition, CODHs are already used in various applications for CO_2_ reduction. Further insight on the functional properties of CODHs can be gained through electrochemical methods. Protein film electrochemistry allows for the in-depth study of the enzyme’s response to external stressors such as changes in pH, applied potential, substrate or inhibitor concentrations and is, therefore, an ideal tool to optimize electrochemical systems, with the goal to enable the transition from fundamental research to technical application. In this review, we described a variety of different applications of CODHs towards CO_2_ reduction to CO, both in purely electrochemical and in photoelectrochemical systems. In recent years, efforts in improving electron transfer, CODH stability and electrode engineering intensified. CO_2_ electrolyzers using CODHs from different biological sources as electrocatalysts were reported with current densities in the range of –mA cm^–2^ and operational stabilities of several hours. This is a promising sign, since apart from energy efficiency, which is inherently given by the CODH’s low overpotential in catalyzing CO_2_ reduction, both the enzyme’s stability and the achievable current density are key factors for rendering future industrial implementation possible. The electrochemical techniques introduced within this review demonstrate how promising CODH enzymes can be for industrial applications. These studies mainly apply CODHs from already cultured microbial strains for CO_2_ reduction on electrodes. Still, this limits our biotechnological possibilities, since the majority of microbes cannot be accessed using culture-dependent methods so far. Therefore, their enzymatic potential remains hidden. Alternatively, the implementation of metagenomics in combination with function-based screening also leads to the identification of truly novel and possibly more active CO_2_ fixing enzymes that could be of industrial importance in the future.

## Data Availability

Not applicable.

## Abbreviations

3HP: 3-Hydroxypropionate
3HP/4HB: 3-Hydroxypropionate/4-hydroxybutyrate
5,10-mTHF: 5,10-Methylene tetrahydrofolate
ACS: Acetyl-CoA synthase
Ag: Silver
ATP: Adenosine triphosphate
Au: Gold
CBB: Calvin–Benson–Bassham
CdS: Cadmium sulfide
CdSe: Cadmium selenide
CFeSP: Corrinoid iron–sulfur protein
CH_4_: Methane
CO: Carbon monoxide
CO_2_: Carbon dioxide
CO_2_RR: Carbon dioxide reduction reaction
CODH: Carbon monoxide dehydrogenase
Cox: Carbon monoxide:acceptor oxidoreductase
Cu: Copper
DC/HB: Dicarboxylate/4-hydroxybutyrate
DET: Direct electron transfer
EcH: Energy converting hydrogenase
FAD: Flavin–adenine–dinucleotide
Fe: Iron
FDH: Formate dehydrogenase
GAP: Glyceraldehyde-3-phosphate
H^+^: Protons
H_2_: Hydrogen
HCO_3_^−^: Bicarbonate
MET: Mediated electron transfer
Mo: Molybdenum
Na: Sodium
NADPH: Nicotinamide adenine dinucleotide
Ni: Nickel
NO_3_^−^: Nitrate
O_2_: Oxygen
PFE: Protein film electrochemistry
rGly: Reductive glycine
RnF: Energy-converting NADH:Fdox oxidoreductase
roTCA: Reverse oxidative tricarboxylic acid
rTCA: Reductive tricarboxylic acid
RubisCO: Ribulose-1,5-bisphosphate-carboxylase/-oxygenase
RuBP: Ribulose-1,5-bisphosphate
SHE: Standard hydrogen electrode
SO_4_^2^^−^: Sulfate
SRB: Sulfate reducing bacteria
TCA: Tricarboxylic acid
TiO_2_: Titanium dioxide
TOF: Turnover frequency
WL: Wood–Ljungdahl
